# RBC-hitchhiking PLGA nanoparticles loading β-glucan as a delivery system to enhance *in vitro* and *in vivo* immune responses in mice

**DOI:** 10.3389/fvets.2024.1462518

**Published:** 2024-09-16

**Authors:** Sheng Li, Yao Wang, Qianqian Liu, Feng Tang, Xinnan Zhang, Shuyao Yang, Qiran Wang, Qian Yang, Shanshan Li, Jie Liu, Lu Han, Yi Liao, Xuemei Yin, Jing Fan, Haibo Feng

**Affiliations:** ^1^College of Animal Husbandry and Veterinary Medicine, Southwest Minzu University, Chengdu, Sichuan, China; ^2^Institute of Qinghai-Tibetan Plateau, Southwest Minzu University, Chengdu, Sichuan, China; ^3^College of Pharmacy, Chengdu University, Chengdu, China

**Keywords:** RBC-hitchhiking, β-glucan, immunization, nanoparticle, polysaccharide

## Abstract

Red blood cells (RBCs) naturally trap some bacterial pathogens in the circulation and kill them by oxidative stress. Following neutralization, the bacteria are presented to antigen-presenting cells in the spleen by the RBCs. This ability of RBCs has been harnessed to develop a system where they play a crucial role in enhancing the immune response, offering a novel approach to enhance the body’s immunity. In this work, a conjugate, G-OVA, was formed by connecting β-glucan and OVA through a disulfide bond. Poly (lactic-co-glycolic acid) (PLGA) was then employed to encapsulate G-OVA, yielding G-OVA-PLGA. Finally, the nanoparticles were adsorbed onto RBCs to develop G-OVA-PLGA@RBC. The results demonstrated that the delivery of nanoparticles by RBCs enhanced the antibody response to antigens both *in vitro* and *in vivo*. The objective of this study was to investigate the increased immune activity of G-OVA-PLGA nanoparticles facilitated by RBCs transportation and to elucidate some of its underlying mechanisms. These findings are anticipated to contribute valuable insights for the development of efficient and safe immune enhancers.

## 1 Introduction

Red blood cells (RBCs) have a double-concave disc-shaped morphology and lack organelles in maturity. With a life cycle of approximately 40 days in mice, the distinctive characteristics of RBCs, including plasticity and robustness of their membrane and cytoskeleton, the absence of nuclei and organelles, and specific molecular characteristics on their surface, collectively grant them unparalleled durability, tensile strength, and deformability (1). Furthermore, RBCs, being integral in the transport of oxygen and carbon dioxide in the bloodstream, manifest outstanding biocompatibility. The aforementioned properties position RBCs as promising candidates for drug delivery systems in disease treatment. Notably, the utilization of RBCs in therapeutic interventions holds an advantageous position over alternative gene or cell therapies in terms of safety (2). The inherent features of RBCs not only make them suitable for effective drug delivery but also emphasize their potential as a safe option for therapeutic uses.

RBCs drug loading involves both internal and external drug loading, and it includes drug loading onto genetically engineered RBCs and RBC-based artificial antigen-presenting cells (3–5). There are two main methods for loading drugs into RBCs: osmosis and endocytosis (6). Osmosis involves using physical methods to create transient pores in the RBC membrane, and the pore size of such transient pores is small, only for small molecules less than 50 nm, including enzymes, antigens, dexamethasone, and nanomedicines (7–10). In addition, endocytosis is the process by which drugs are loaded into cells by RBCs through endocytosis, including primaquine, chlorpromazine, hydrocortisone, and bupivacaine (4, 11). There are four primary methods for externally loading drugs into RBCs, including chemical binding, RBC-hitchhiking, cluster-specific binding of determinants on the surface of the RBC membrane, and genetic modification of reticulocytes (1). Furthermore, the RBC-hitchhiking technique is a popular and convenient method of external drug delivery. In this technique, the drug is adsorbed onto the RBC membrane, and the drug-loaded cells are introduced into the body through intravenous injection (12).

β-glucan is a polysaccharide composed of D-glucopyranose residues bonded by β-glycosidic linkages. It can be extracted from the cell walls of mushrooms, yeast, oats, barley, algae, and bacteria. β-glucans from different sources have varying primary structures and conformations (13, 14). The biological activity of β-glucan is intricately linked to its primary structure and conformation. For example, mushroom polysaccharide consists of β-(1,3)-glucan, which demonstrates notable anti-tumor activity due to its β-helical conformation (15).

β-glucan has been extensively studied for its immunomodulatory activity (16, 17). It has also been investigated as an anti-infective vaccine adjuvant and vaccine delivery system. β-glucan induces trained immunity by modifying histones on the promoter of human monocyte genes. Monocytes, after undergoing training, display a protective effect against *Mycobacterium tuberculosis* (Mtb) infection (18). In addition to its application in anti-tuberculosis strategies, β-glucan finds common use in the aquaculture and food industries due to its immunostimulatory properties. Studies have indicated that incorporating β-1,3 or 1,6-glucan and model vaccines into the diets of salmon can lead to alterations in major cytokines such as IFN-γ and IL-12. This dietary inclusion of β-glucan enhances both congenital and acquired immune responses. In addition, β-Glucan can attract dendritic cells, amplify the recognition of tumor antigens by these cells, and boost the expression and responsiveness of Th1-biased cytokines within the tumor microenvironment (19).

**Figure 1:**
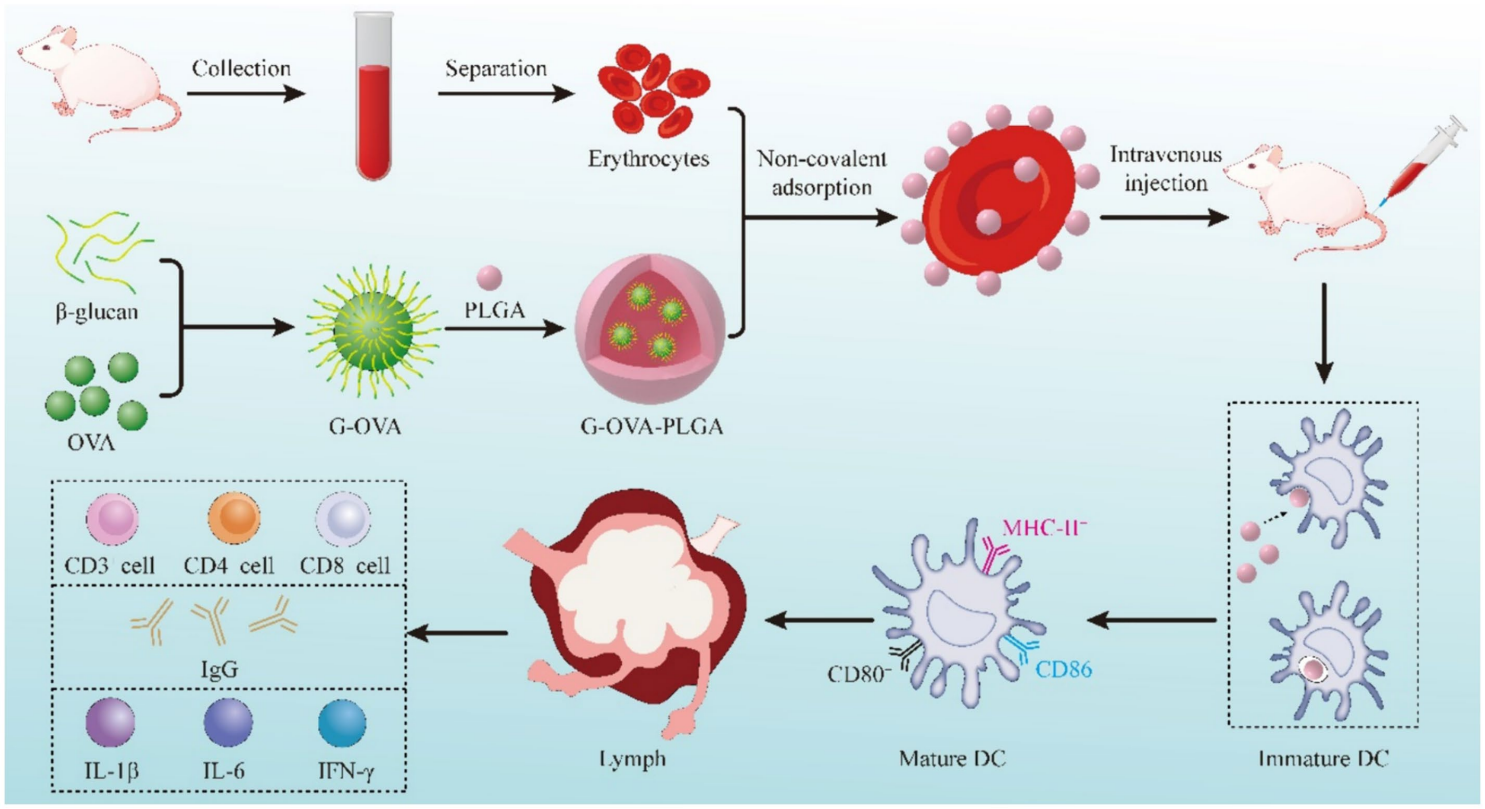
Schematic diagram of preparing the G-OVA-PLGA@RBC delivery system.

Here, we prepared PLGA-coated β-glucan nanoparticles with OVA coupling products and delivered the nanoparticles adsorbed on the surface of erythrocytes for cellular and humoral immunity. Figure 1 shows the fabrication process of the nanoparticles, as well as the *in vivo* and *ex vivo* tests.

## 2 Materials and methods

### 2.1 Materials

The yeast β-glucan was supplied by Angel Yeast Co., Ltd. (Shanghai, China). OVA, 3-2-pyridyldithiopropionylhydrazine (PDPH), N, N-dimethylformamide (DMF) were procured from Sigma-Aldrich (USA). Fluorescent antibodies (PE-CD80^+^, PE-CD4^+^, PE-CD8^+^, PE-CD86^+^, PE-MHC-II^+^, FITC-CD11c^+^ and FITC-CD3^+^) were supplied by eBioscience (CA, USA). Nitric Oxide Assay Kit was supplied by US Everbright Co., Ltd. (USA). TRNzol Universal Tiangen Biotech Co., Ltd. (Beijing, China). PrimeScript™ RT reagent kit and TB Green® Premix Ex Taq™ II kit were purchased from Takara Biotechnology Co., Ltd. (Beijing, China). Cy5.5 fluorescent dye was obtained from Xi’an Qiyue Biology Co., Ltd. (Xian, China). Goat Anti-Mouse IgG/HRP and DID were obtained from Solarbio Technology Co., Ltd. (Beijing, China). Mouse RAW264.7 macrophages was obtained from SUNNCELL Co., Ltd. (Wuhan, China).

### 2.2 Preparation and characterization of G-OVA-PLGA

**Figure 2:**
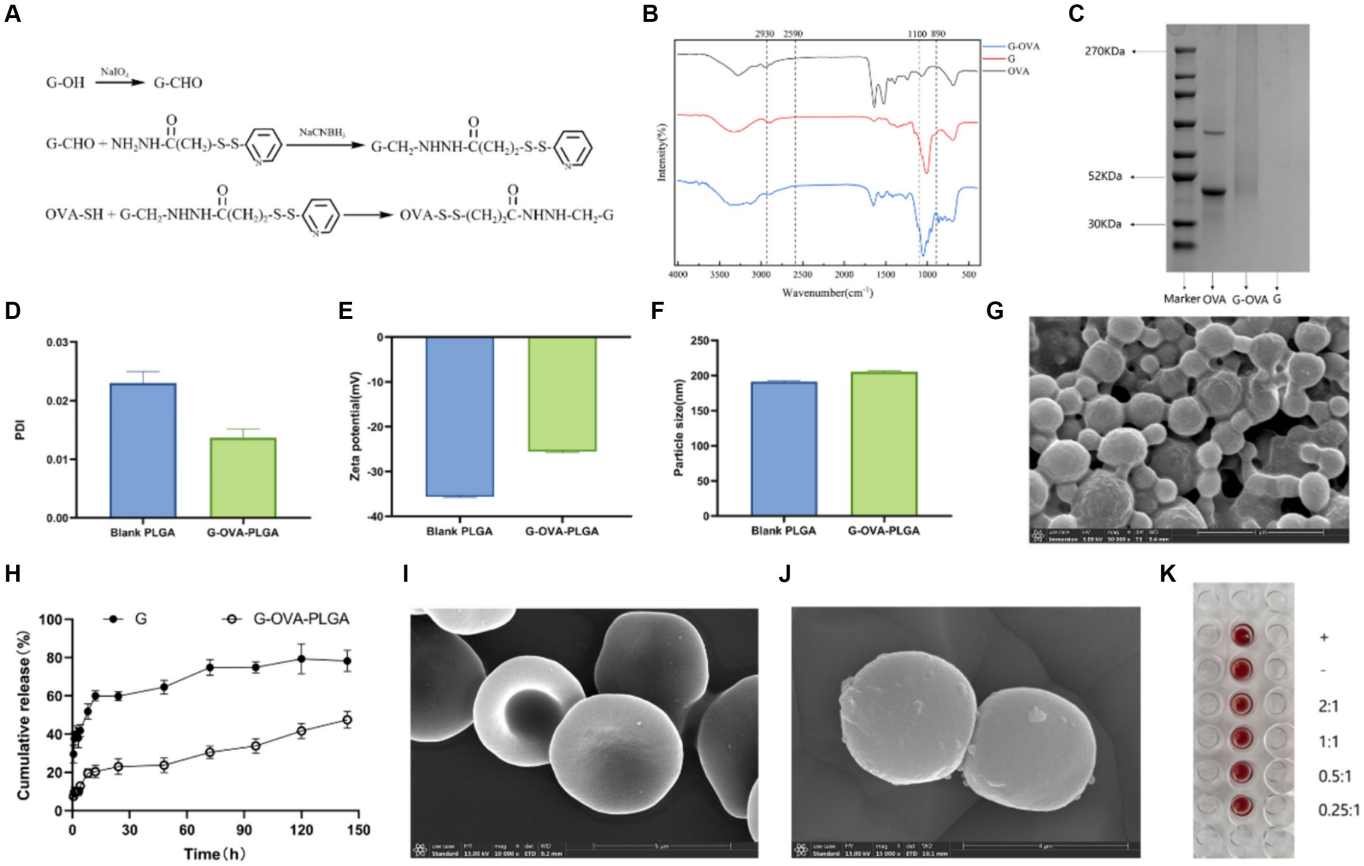
Characterization of G-OVA-PLGA. **(A)** Schematic representation of the preparation of β-glucan and OVA conjugate linked via disulfide bonds. **(B)** FTIR spectrum of G-OVA. **(C)** SDS-PAGE gel electric swimming. **(D)** G-OVA-PLGA nano-grains polydispersity index (PDI). **(E)** Particle size. **(F)** Zeta potential. **(G)** Scanning electronic micrographs of G-OVA-PLGA nanoparticles. **(H)** *In vitro* drug release curve. **(I)** Blank RBC scanning electron micrograph (SEM). **(J)** RBC-hitchhiking nanoparticles scan electron microscope (SEM). **(K)** Agglutination test.

Disulfide bonds were employed in the preparation of G-OVA to connect β-glucan and OVA (20). In a simplified process, β-glucan underwent oxidation initially. A 5 mg/mL β-glucan solution was thoroughly mixed with a 100 mM sodium periodate solution, and the reaction was continued at room temperature for 20 min in the absence of light. Subsequently, the β-glucan derivatives with aldehyde groups were obtained by full dialysis into PBS buffer (pH = 7.2) using a dialysis bag with a molecular weight cut-off of 7 k Da. A solution of 2-3-pyridyldithiopropionylhydrazine (PDPH), obtained by dissolving 2.5 mg PDPH in N, N-dimethylformamide (DMF), was then mixed with 10 mg of sodium cyanoborohydride (NaBH_3_CN) dissolved in PBS buffer (pH = 7.2). This mixture was combined with oxidized β-glucan and allowed to react at 4°C for 12 h. Subsequently, dialysis was performed to eliminate any unreacted PDPH. Finally, OVA (0.5 mg/mL, 2 mL) was added, and the reaction proceeded at 4°C for 12 h, resulting in the formation of G-OVA comprising β-glucan and OVA linked by disulfide bonds. The specific chemical principle has been illustrated in Figure 2A. G-OVA was characterized using Fourier Transform Infrared Spectroscopy and sodium dodecyl sulfate polyacrylamide gel electrophoresis (SDS-PAGE).

Subsequently, G-OVA-PLGA nanoparticles were generated through the nanoprecipitation method (21). A mixture of 2 mg G-OVA and 50 mg PLGA in 2 mL acetone was blended to form an aqueous solution. This suspension was stirred with a magnetic stirrer and introduced dropwise into a solution containing 1% w/v F68. Following the complete volatilization of the organic solvent, the resulting suspension underwent centrifugation (12,000 × g, 4°C, 10 min), retaining only the precipitate. Blank nanoparticles and OVA-loaded nanoparticles were also prepared and dried using the same methodology. Particle size, polydispersity index, and zeta potential were determined using a zeta potential analyzer (Zetasizer Nano ZS). Additionally, the morphological characteristics of G-OVA-PLGA nanoparticles after freeze-drying were observed by scanning electron microscopy (S-4800). The encapsulation efficiency of nanoparticles to G-OVA was detected by microcolumn centrifugation (22). The calculation formula is as follows:


EE(%)=Ce/Ct×100

Where EE is the encapsulation efficiency, Ce is the count of G-OVA encapsulated, and Ct represents the total count of G-OVA.

The *in vitro* drug release kinetics was observed by the dialysis method (23). In a simulated body fluid environment, G-OVA-PLGA nanoparticles and β-glucan suspension were separately enclosed in dialysis bags and subsequently immersed in 0.1% Tween 80 PBS (pH = 7.4). The dialysate was collected at specific time intervals, and an equivalent volume of fresh permeation medium was promptly replenished. The release of β-glucan was then assessed using the phenol-sulfuric acid method.

### 2.3 Preparation and characterization of G-OVA-PLGA@RBC

Whole blood was collected from the retro-orbital venous plexus of mice, and centrifuged to separate plasma and erythrocytes after leaving on ice for 30 min. The RBCs were washed with pre-cooled PBS, and the washed RBCs were resuspended with 10% hematocrit. The RBCs underwent a 30 min treatment with nanoparticles at 37°C, followed by the separation of excess nanoparticles through centrifugation (24). Confirmation of nanoparticle adsorption onto the RBC surface was achieved using scanning electron microscopy. In brief, the samples were immersed in 4% glutaraldehyde for 1 h, rinsed with PBS to eliminate unreacted glutaraldehyde, and subsequently dehydrated in a series of ethanol washes with increasing concentrations (50, 60, 70, 80, 90, 100%). The samples were then suspended in 100% ethanol and dried using a critical point dryer for imaging (25). Finally, the samples were observed using a scanning electron microscope (S-4800).

The effect of nanoparticles on RBC was evaluated by agglutination test. G-OVA-PLGA nanoparticles were mixed with 10% RBCs in the ratios of 2:1, 1:1, 0.5:1, 0.25:1 and then dispensed into a 96-well U-plate, which was left at 37°C for 1 h.

### 2.4 Effect of G-OVA-PLGA on the viability of mouse RAW264.7 macrophages

The mouse macrophage activity was assessed using the CCK-8 assay to evaluate the potential toxicity of G-OVA-PLGA nanoparticles on mouse macrophages. RAW 264.7 cells were seeded in 96-well plates at the density of 1 × 10^4^ cells/well and incubated for 12 h. Solutions containing G-OVA-PLGA nanoparticles, blank PLGA nanoparticles, and β-glucan were added at various concentrations (15.625, 31.25, 62.5, 125, 250, 500, and 1,000 μg/mL) to the cell culture plates. Following the CCK-8 kit instructions, OD readings were recorded at 450 nm. The calculation formula is as follows:


Cellsurvivalrate=[(As−Ab)/(Ac−Ab)]×100%

Where As is the absorbance of the experimental well, Ac: is the absorbance of the control well. and Ab: is the absorbance of the blank well.

### 2.5 Effect of G-OVA-PLGA on NO production by mouse RAW264.7 macrophages

RAW264.7 cells were inoculated into 96-well plates at the density of 4 × 10^4^ cells/well and cultured for 24 h. After the addition of different concentrations of lipopolysaccharide (LPS), β-glucan (G), and G-OVA-PLGA nanoparticles (500, 250, and 125 μg/mL), the incubation was continued for 24 h. Then take the supernatant and measure the level of Nitric oxide (NO) according to the instructions given by Nitric Oxide Assay Kit. Briefly, 100 μL of culture supernatant was taken, and 50 μL/well of Griess reagent I and 50 μL/well of Griess reagent II were added in sequence. The absorbance was measured at 540 nm, and the concentration of NO was calculated according to the standard curve.

### 2.6 Effect of G-OVA-PLGA nanoparticles on the phenotype and function of RAW264.7 macrophages

Flow cytometry was employed to quantify the surface expression of macrophage molecules. RAW264.7 cells suspension (5 × 10^4^ cells/well) was plated on a 6-well plate for 24 h. G-OVA-PLGA nanoparticles, G, and LPS with a concentration of 250 μg/mL were added to the wells for 24 h, and three repeated wells were made for each group. Following treatment, these cells were stained with PE-CD80^+^ and PE-CD86^+^ in the dark and subsequently analyzed using flow cytometry. Statistical analysis was carried out using Flow Jo 10.4 software.

### 2.7 Study on the uptake of G-OVA-PLGA nanoparticles by mouse RAW264.7 macrophages

The G-OVA-PLGA nanoparticles were stirred with FITC (Fluorescein 5-isothiocyanate) for 12 h, dialyzed in the dark to remove excess FITC, and then freeze-dried under light protection into a powder. FITC and OVA were mixed in a ratio of 1:2. Following cell adhesion, a mixture of OVA, β-glucan, and FITC-labeled G-OVA-PLGA nanoparticles was introduced and allowed to incubate for 12 h. Subsequently, the phagocytosis rate was evaluated using flow cytometry (CyFlow Cube8, Sysmex, Germany). In addition, the cells were cultured on cell slides, fixed with 4% paraformaldehyde for 20 min, and then stained with DID (1,1’-Dioctadecyl-3,3,3′,3′-tetramethylindodicarbocyanine perchlorate) for 20 min, washed three times with PBS, and then stained with DAPI (4′,6-diamidino-2-phenylindole) for 20 min, and finally mounted with 90% glycerol, and the phagocytosis was observed under an inverted fluorescence microscope. Statistical analysis was carried out using Flow Jo 10.4 software.

### 2.8 Animal experiments

All animal experiments were carried out following the internationally recognized principles mentioned in the laboratory animal feeding guidelines issued by the Chinese government and approved by Southwest Minzu University for Nationalities. ICR female mice aged 7–8 weeks were supplied by Chengdu Dashuo Experimental Animal Co., Ltd.

To examine the humoral and cellular immune responses induced by RBC-hitchhiking nanoparticles in healthy mice. Female ICR mice aged 7–8 weeks received injections of saline, OVA, G-OVA-PLGA, OVA-PLGA@RBC, and G-OVA-PLGA@RBC. The OVA concentration was maintained at 170 μg/mL, and the β-glucan concentration was 300 μg/mL. Injections were administered on days 0, 7, 14, and 21. As a positive control, complete Freund’s adjuvant (with an OVA concentration of 170 μg/mL) was subcutaneously injected.

### 2.9 Effect on dendritic cell maturation in the spleen

48 h after the first immunization, 3 mice were randomly selected from each group, and the spleens of the experimental mice were collected, ground and passed through a 300-mesh cell sieve, and red blood cell lysis buffer was added to make a single cell suspension, which was stained for 30 min under light-proof conditions. Flow cytometry (CyFlow Cube8, Sysmex, Germany) was used to detect the presence of PE and FITC-labeled CD11c^+^, MHC-II^+^, CD80^+^, and CD86^+^ surface markers in dendritic cells, and the data were finally statistically analyzed using FlowJo10.4 software.

### 2.10 ELISA for antibody determination

On days 7, 14, 21, and 28 after immunization, 3 mice were randomly selected from each group, and blood from test mice was taken through an orbital vein, followed by the separation of serum. An indirect ELISA was used to detect OVA-specific IgG antibody levels in serum. Briefly, OVA solution (5 μg/mL) was added to a 96-well plate, incubated at 4°C for 17 h, washed 3 times with washing solution, 1% skimmed milk was added to block, and after removing the blocking solution, diluted serum was added to incubate for 1 h. Next, Goat anti-mouse IgG/HRP was added, and chromogenic solution and stop solution were added, and finally the absorbance of the sample was measured at 450 nm using a microplate reader.

### 2.11 Effect on splenic T-lymphocyte subsets

On the 28th day after immunization, 3 mice were randomly selected from each group, the spleens of the experimental mice were collected, and red blood cell lysis buffer was added to make a single cell suspension. The cells were incubated with PE-CD4^+^, PE-CD8^+^ and FITC-CD3^+^ antibodies for 30 min under light-proof conditions, and then resuspended in 1 mL of PBS. Flow cytometry (CyFlow Cube8, Sysmex, Germany) was used to analyze the expression levels of CD4^+^ and CD8^+^ in T lymphocytes, and finally FlowJo10.4 software was used for statistical analysis.

### 2.12 Effect of G-OVA-PLGA@RBC on the spleen index and thymus index in mice

On the 28th day, 3 mice were randomly selected from each group, the mice were euthanized, and their spleens and thymuses were harvested and weighed. The spleen and thymus indexes were calculated as follows:

$\begin{array}{c} \mathrm{Immune organ index}\;\left(\mathrm{mg}/g\right)=\mathrm{immune organ mass}\;\left(\mathrm{mg}\right)/\\ \mathrm{body weight}\;\left(g\right) \end{array}$

### 2.13 Analysis of spleen cytokines

Three mice were randomly selected from each group, and the expression levels of IL-1β, IL-6, and IFN-γ cytokine in the spleen were measured by Quantitative Real-time PCR. Spleen total RNA was isolated through the Trizol method. The RNA was then transcribed into cDNA utilizing PrimeScript™ RT reagent kit and subjected to real-time quantitative fluorescence PCR using a TB Green® Premix Ex Taq™ II kit.

### 2.14 Study of biological distribution of G-OVA-PLGA@RBC

To investigate the distribution of drugs in mice, we used an *in vivo* optical imaging system (IVIS Lumina III, Perkin Elmer). 27 healthy mice were randomly divided into three groups, 9 in each group. OVA, G-OVA-PLGA, and G-OVA-PLGA@RBC were labeled with Cy5.5 fluorescent dye and administered through tail vein injection. The distribution of the drugs in mice was monitored through fluorescence imaging at 0.5 h, 6 h, and 24 h post-injection.

### 2.15 Histopathological analysis

On the 28th day, the key organs of test mice, including the heart, liver, spleen, lung, and kidney, were collected. Tissue morphology was observed under the microscope using hematoxylin–eosin staining.

### 2.16 Blood routine analysis

To evaluate the safety, on the 28th day after immunization, 3 mice were randomly selected from each group to collect blood from the orbital vein, and the blood was analyzed using a routine Blood analyzer (Mindray BC-5000Vet).

### 2.17 Statistical analysis

Statistical analysis was performed using SPSS 23.0. Data were analyzed by one-way ANOVA; multiple comparisons were performed using the Least Significant Difference method, the Waller-Duncan method, and the Tukey method. Data has been presented as mean ± standard deviation. A significance level of *p* < 0.05 was considered statistically significant.

## 3 Results

### 3.1 Characterization of G-OVA-PLGA nanoparticles

G-OVA-PLGA nanoparticles were characterized by FTIR spectroscopy. The results are shown in the Figure 2B, both G and G-OVA showed characteristic peaks at 890 cm^−1^ (C-H variable angle vibration), 950 cm^−1^ (O-H bending vibration), 1,100 cm^−1^ (C-O stretching vibration), 2,849 cm^−1^ (-CH_2_ symmetric stretching vibration) and 2,930 cm^−1^ (-CH_2_ asymmetric stretching vibration). OVA exhibits an absorption peak for hydrophobic (S-H) stretching vibration at 2,590 cm^−1^. Notably, this peak was absent in G-OVA, suggesting the coupling of the aldehyde group (-CHO) in oxidized β-glucan with the hydrophobic group (S-H) in OVA through disulfide bonds (20). Moreover, OVA has a molecular weight of approximately 44 k Da, but upon coupling with β-glucan, the molecular weight of G-OVA exceeded that of OVA. The characteristics of OVA and G-OVA were assessed using SDS-PAGE, as illustrated in Figure 2C. The appearance of a higher band for G-OVA compared to OVA suggests an increase in molecular weight due to the successful coupling of β-glucan with OVA. Importantly, the β-glucan lane did not show any band. Polysaccharides like β-glucan, being non-proteinaceous molecules, do not respond well to the denaturing conditions of SDS-PAGE, and their large, complex structures can hinder their migration through the gel. As a result, β-glucan may not produce a distinct band on the gel. This observation further supports the successful coupling of β-glucan with OVA. The lack of a band in the β-glucan lane supports the notion that β-glucan is indeed part of the G-OVA complex rather than existing separately in the sample. In conclusion, these findings confirm the effective conjugation of β-glucan with OVA.

Drug-loaded biodegradable PLGA nanoparticles were prepared by the nanoprecipitation method. The diameter of the drug-loaded PLGA nanoparticles was 202 ± 2.4 nm, slightly greater than that of the blank nanoparticles (Figure 2F). The surface charge of the blank nanoparticles was −35.6 ± 0.81Mv (Figure 2E), and the addition of G-OVA reduced the surface charge of the drug-loaded nanoparticles (−25.6 ± 1.5 mV). The encapsulation efficiency was estimated to be 55.12 ± 4.1%. Scanning electron microscopy (SEM) was employed to examine the morphology of the nanoparticles. The SEM image depicted shows that both blank and drug-loaded PLGA nanoparticles display a spherical shape (Figure 2G).

To assess the release characteristics of G-OVA-PLGA nanoparticles, we examined their release in the medium (Figure 2H). During the initial 24 h, both β-glucan and nanoparticles displayed a substantial increase in release rate, with β-glucan showing a notably higher initial release rate than the nanoparticles. Over the subsequent 120 h, the cumulative release rate of β-glucan reached 82.84%, whereas that of the nanoparticles was 43.18%. In summary, the current data indicate that G-OVA-PLGA nanoparticles demonstrate considerably slower *in vitro* release characteristics compared to β-glucan.

### 3.2 Characterization of G-OVA-PLGA@RBC

The assembly of drug-loaded G-OVA-PLGA nanoparticles on mouse RBCs was observed using SEM. As depicted in Figures 2I,J, the drug-loaded RBCs retained their characteristic double concave shape, suggesting minimal damage caused by the nanoparticles. Furthermore, the agglutination test demonstrated that RBCs incubated with the nanoparticles did not show an agglutination reaction (Figure 2K), implying the suitability of G-OVA-PLGA@RBC for injection into the body. In conclusion, these findings confirm the successful preparation of G-OVA-PLGA@RBC.

### 3.3 Effect of G-OVA-PLGA on the viability of mouse RAW264.7 macrophages

**Figure 3:**
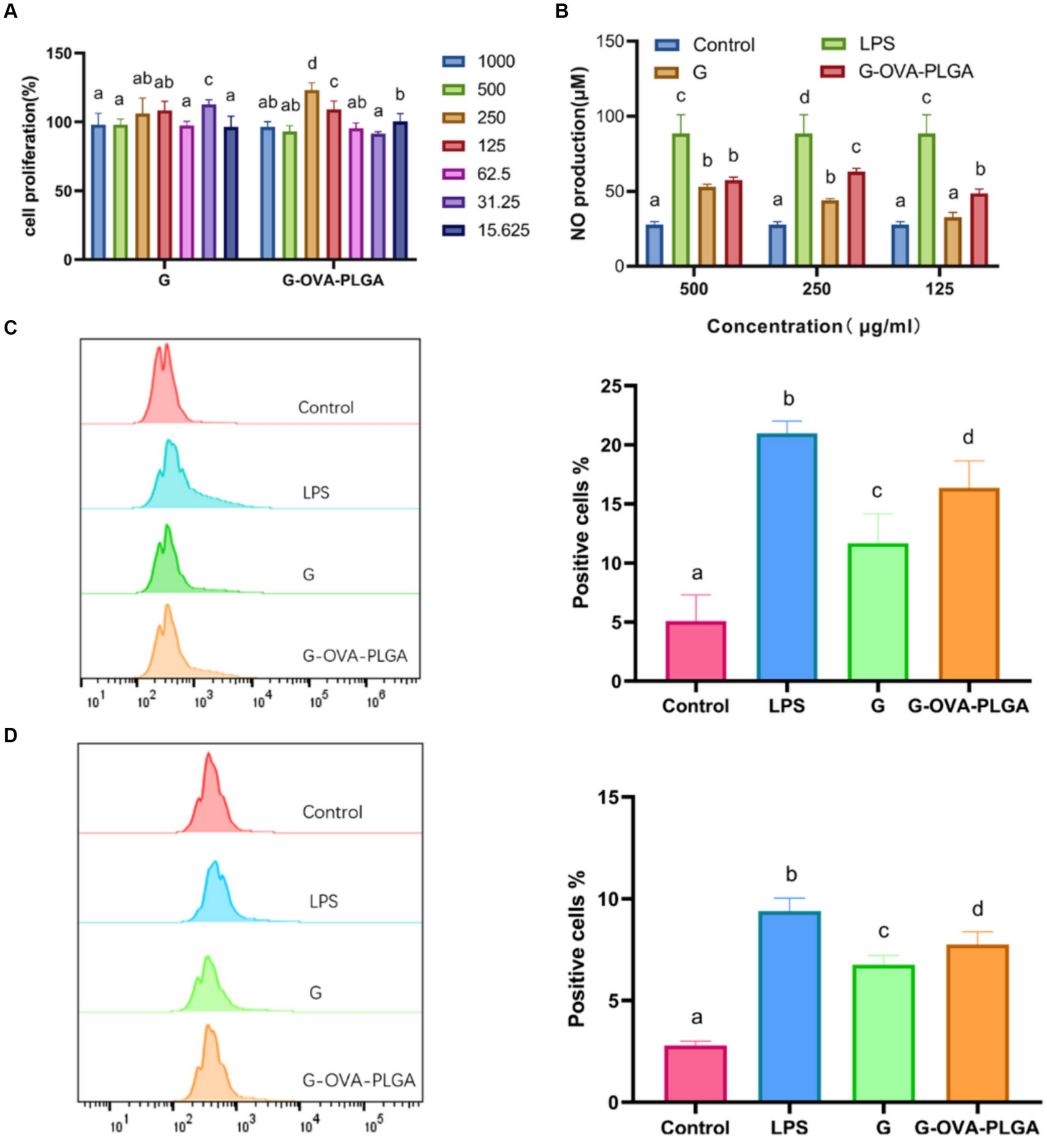
**(A)** Effect of G-OVA-PLGA nanoparticles on macrophage activity. **(B)** NO secretion levels. The expression levels of CD80 ^+^ **(C)** and CD86 ^+^ **(D)** on the surface of macrophages detected by flow cytometry. Different letters (a–d) represent statistically significant differences (*p* < 0.05).

The impact of various nanoparticle concentrations on the relative survival of RAW 264.7 was examined. As depicted in Figure 3A, the relative survival rates of all nanoparticle groups exceeded 82%. This indicates that nanoparticles within the concentration range of 15.625 to 1,000 μg/mL showed no significant toxic effects on macrophages RAW 264.7.

### 3.4 Effect of G-OVA-PLGA on NO production by mouse RAW264.7 macrophages

NO plays a crucial role in the regulation of both innate and acquired immunity and is widely distributed in various tissues and organs (26). As shown in Figure 3B, within the concentration range of 125 μg/mL to 500 μg/mL, G-OVA-PLGA nanoparticles exhibited a significant enhancement in NO secretion compared to blank PLGA. Specifically, at concentrations of 125 μg/mL and 250 μg/mL, G-OVA-PLGA nanoparticles demonstrated a noteworthy increase in NO secretion compared to β-glucan (*p* < 0.05). In summary, G-OVA-PLGA nanoparticles can stimulate an elevation in NO secretion in RAW 264.7 macrophages.

### 3.5 Effect of G-OVA-PLGA nanoparticles on the phenotype and function of mouse RAW 264.7 macrophages

Upon appropriate stimulation, macrophages can be activated into different inflammatory states, including classical activation (M1) and selective activation (M2) (27). Our study focused on the impact of G-OVA-PLGA on the surface expression of co-stimulatory molecules CD80^+^ and CD86^+^ in macrophages RAW 264.7 which was assessed by flow cytometry. As demonstrated in Figures 3C,D, the stimulation with G-OVA-PLGA significantly enhanced the expression levels of the macrophage surface molecules CD80^+^ and CD86^+^, indicating the potential of G-OVA-PLGA to modulate the phenotype of macrophages. In summary, G-OVA-PLGA can induce the activation of macrophages into an M1-type inflammatory state.

### 3.6 Study of G-OVA-PLGA nanoparticles uptake by RAW 264.7 macrophages

**Figure 4:**
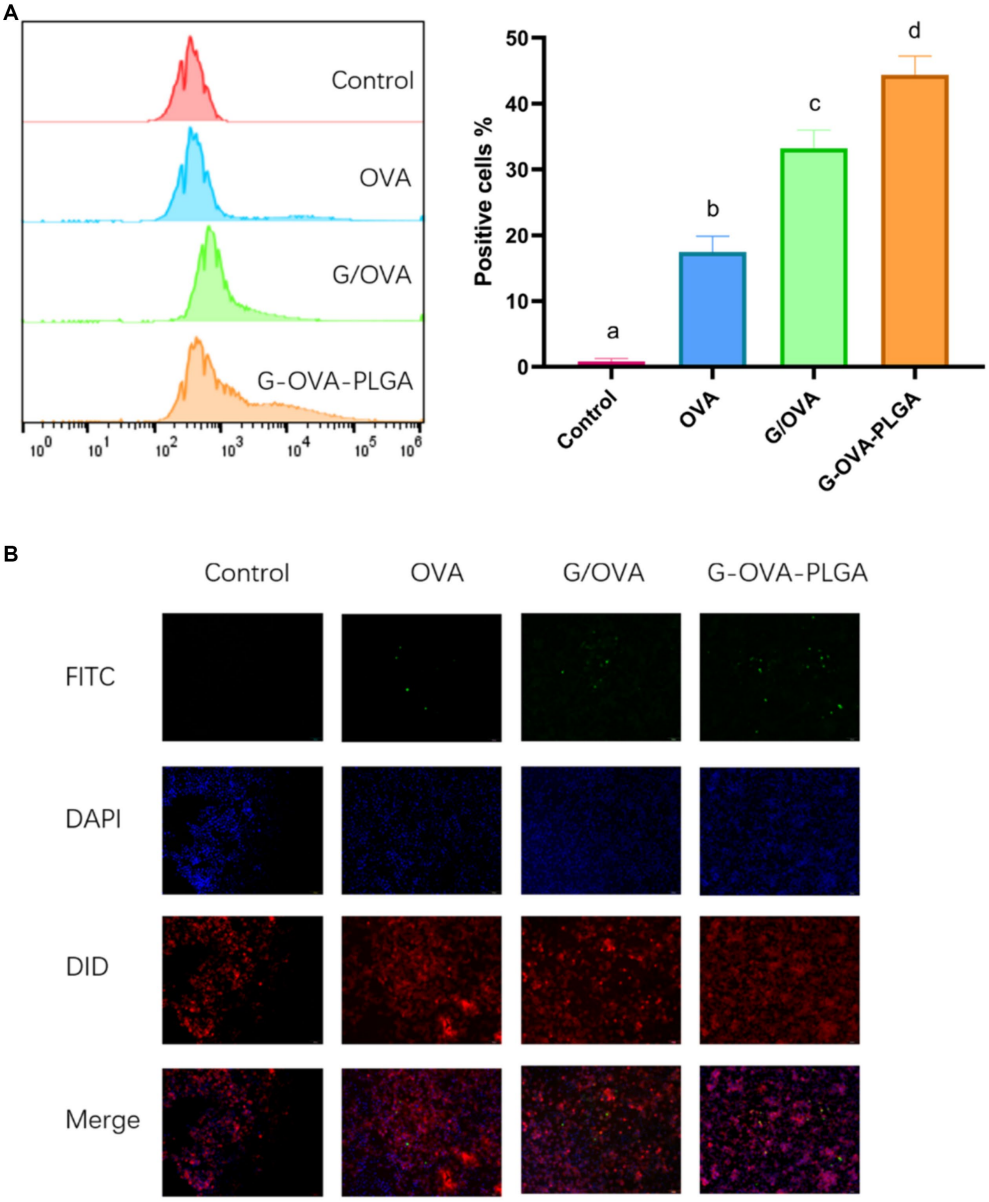
Effect of G-OVA-PLGA on the phagocytic activity of macrophages. **(A)** Flow histogram and quantitative analysis chart of fluorescence intensity. **(B)** Uptake of G-OVA-PLGA by macrophages observed by an inverted fluorescence microscope. Different letters (a-d) represent statistically significant differences (*p* < 0.05).

In this study, the phagocytosis of nanoparticles by macrophages was detected by flow cytometry as well as inverted fluorescence microscopy. As depicted in Figure 4B, the fluorescence intensity of G-OVA-PLGA nanoparticles was significantly greater than that of OVA and G/OVA, suggesting an enhanced uptake of OVA by macrophages facilitated by G-OVA-PLGA nanoparticles. Additionally, the detection of FITC-labeled drugs through flow cytometry allowed for the quantification of macrophage phagocytic ability, expressed as the percentage of positive fluorescence within macrophages. As shown in Figure 4A, the positive rate of G-OVA-PLGA nanoparticles was significantly higher than that of OVA and G/OVA (*p* < 0.05), which was consistent with the results obtained via inverted fluorescence microscopy. In summary, G-OVA-PLGA nanoparticles can activate macrophages and improve their ability to engulf antigens.

### 3.7 Effect on dendritic cell maturation in the spleen

**Figure 5:**
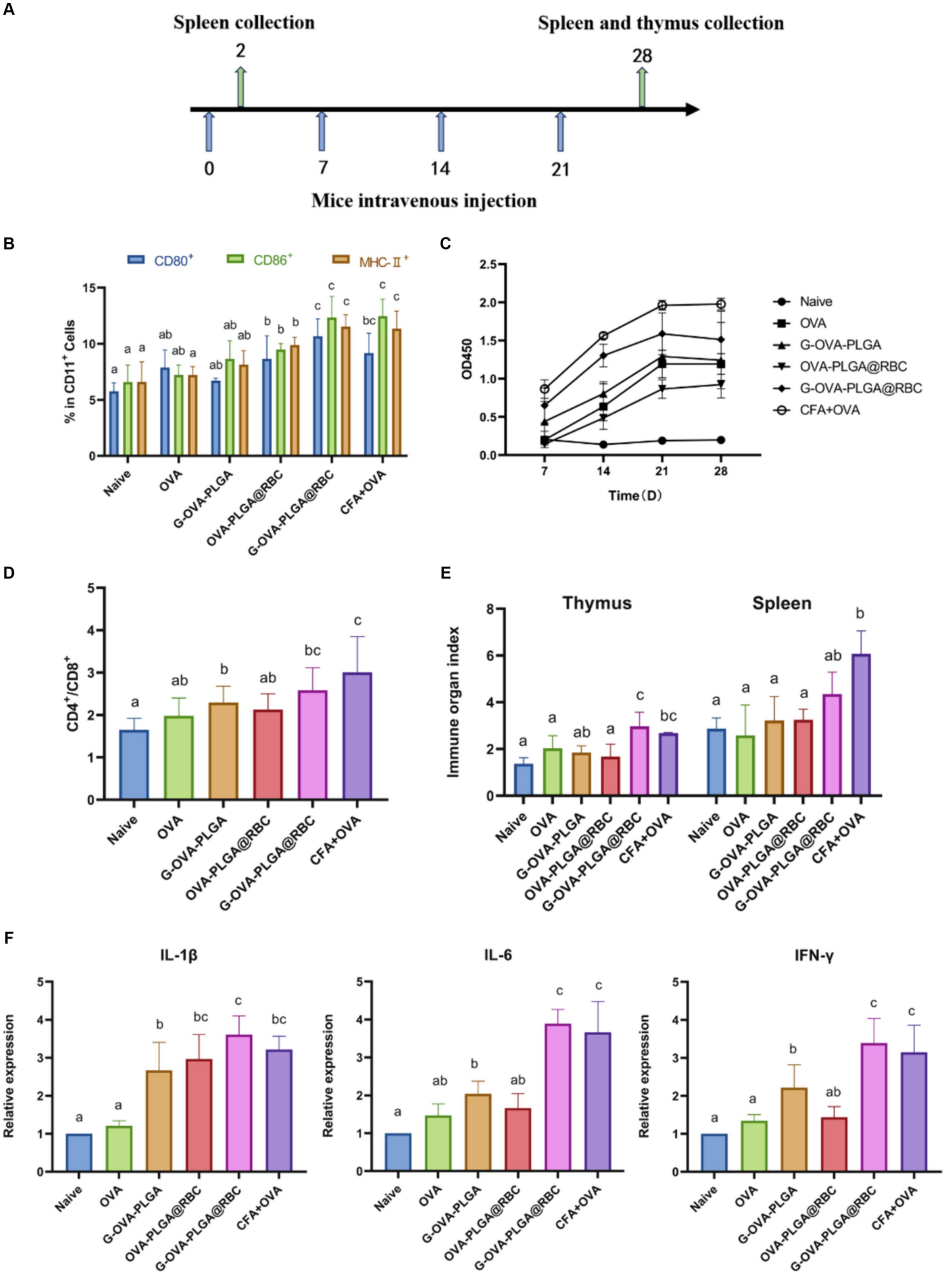
**(A)** Schematic diagram of drug treatment time. **(B)** Percentage of CD80^+^, CD86^+^, and MHC-II^+^ cells in the CD11c^+^ DC population. **(C)** OVA-specific IgG antibody. **(D)** The ratio of CD4^+^ and CD8^+^ cells. **(E)** Spleen and thymus organ index. **(F)** Levels of IL-1β, IL-6, IFN-γ. Different letters (a–c) represent statistically significant differences (*p <* 0.05).

The process of immunization of animals through the tail vein has been shown in Figure 5A. Dendritic cells play a pivotal role as antigen-presenting cells, undergoing maturation upon recognizing antigens. Once mature, these dendritic cells present processed antigens to T-lymphocytes, thereby initiating systemic humoral and cellular immunity (28, 29). Dendritic cell maturation was observed in this study by detecting the expression of dendritic cell surface molecules CD80^+^, CD86^+^, and MHC-II^+^. As depicted in Figure 5B, the expression levels of CD80^+^, CD86^+^, and MHC-II^+^ in the G-OVA-PLGA@RBC group were significantly higher compared to the OVA, G-OVA-PLGA, and OVA-PLGA@RBC groups (*p* < 0.05). In conclusion, G-OVA-PLGA@RBC effectively enhanced the expression of CD80^+^, CD86^+^, and MHC-II^+^, promoting the maturation of mouse spleen dendritic cells.

### 3.8 ELISA for antibody determination

To evaluate the impact of immunostimulants on humoral immunity, the levels of serum-specific IgG were assessed using indirect ELISA analysis as shown in Figure 5C. Over the 28 days, the G-OVA-PLGA@RBC group demonstrated significantly higher IgG levels compared to the saline, G-OVA-PLGA, free OVA, and OVA-PLGA@RBC groups (*p* < 0.05). This suggests that G-OVA-PLGA@RBC can elicit more robust humoral immune responses, leading to increased antibody production.

### 3.9 Effect on splenic T-lymphocyte subsets

It can be seen from Figure 5D that the ratio of CD4^+^/CD8^+^ positive cells in the G-OVA-PLGA@RBC group was notably greater than that in other groups (*p* < 0.05), however, it does not surpass that in the CFA + OVA group. In summary, G-OVA-PLGA@RBC can promote the activation of spleen T lymphocytes.

### 3.10 Effect of G-OVA-PLGA@RBC on the spleen index and thymus index in mice

The spleen and thymus indices are among the key reference indicators for assessing immune function. The thymus index and spleen index of the G-OVA-PLGA@RBC group were notably higher than those of OVA, G-OVA-PLGA, and OVA-PLGA@RBC groups (*p* < 0.05) and the results are shown in Figure 5E. In summary, G-OVA-PLGA@RBC up-regulated the immune organ index of mice and promoted immune function.

### 3.11 Analysis of spleen cytokines

IL-1β, IL-6, and IFN-γ are well recognized for their pro-inflammatory effects (30). As shown in Figure 5F, except for the CFA + OVA group, the expression levels of IL-6 and IFN-γ in the spleen of the G-OVA-PLGA@RBC group were significantly higher than those in the other groups (*p* < 0.05). As shown in Figure 5F, the expression of IL-1β in the spleen of G-OVA-PLGA@RBC was notably higher than that of other groups (*p* < 0.05). The results indicated that G-OVA-PLGA@RBC could induce the secretion of IL-1β, IL-6, and IFN-γ and enhance the immune response.

### 3.12 Study of biological distribution of G-OVA-PLGA@RBC

**Figure 6:**
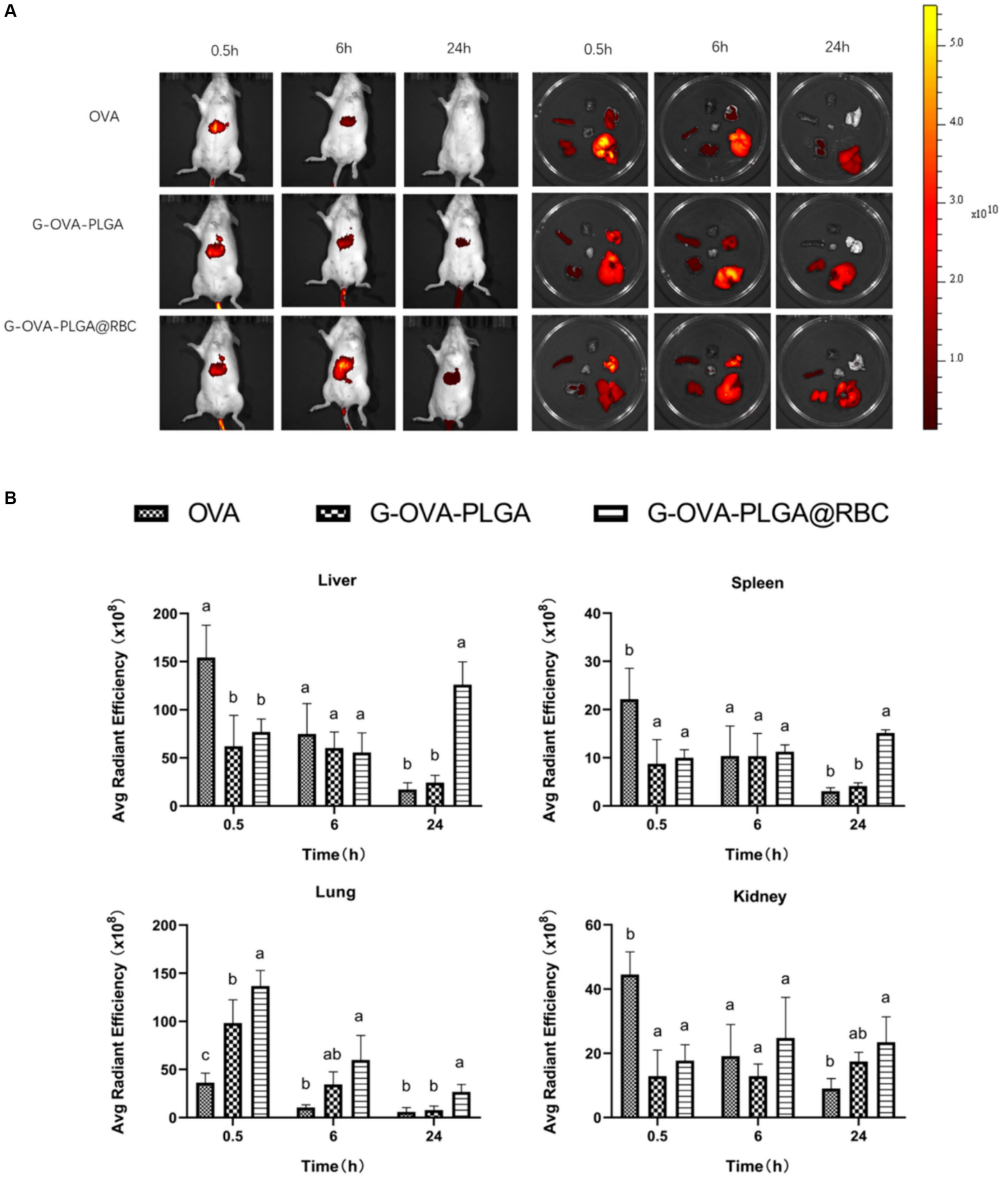
Biodistribution of G-OVA-PLGA@RBC in mice. **(A)** *In vivo* imaging of mice and direct imaging of organs. **(B)** Fluorescence intensity of liver, spleen, lung and kidney. Different letters (a–c) represent statistically significant differences (*p* < 0.05).

To assess the biodistribution of nanoparticles carried by RBCs, the impact of nanoparticle loading on *in vivo* distribution was examined at 0.5 h, 6 h, and 24 h post intravenous injection. As depicted in Figure 6, free OVA primarily accumulated in the liver throughout these three periods, primarily due to phagocytosis by the mononuclear phagocyte system (MPS). During the first 0.5 and 6 h, a significant accumulation of nanoparticles was observed in the lungs of both the G-OVA-PLGA@RBC and G-OVA-PLGA groups. However, the fluorescence intensity in the lungs was significantly higher in the G-OVA-PLGA@RBC group than in the G-OVA-PLGA group (*p* < 0.05). Due to compression and shear stresses experienced by nanoparticles when passing through pulmonary capillaries, those adsorbed on RBCs become dislodged and accumulate in the lungs (24). As the nanoparticles were desorbed from the RBCs, the accumulation of the G-OVA-PLGA@RBC group in the spleen, liver and kidney was significantly higher than that in the lungs over the next 24 h (*p* < 0.05). The nanoparticles were gradually accumulated in the spleen, liver, and kidney during the circulation process, which enhanced the internalization of nanoparticles *in vivo*. Meanwhile, the accumulation of G-OVA-PLGA@RBC group in the spleen, liver, and lung was significantly higher than that of the G-OVA-PLGA group (*p* < 0.05). This suggests that the metabolism of the G-OVA-PLGA@RBC group was slower than that of the G-OVA-PLGA group. In conclusion, the G-OVA-PLGA@RBC group initially accumulates in the lungs and subsequently in the spleen and liver after 24 h, inducing a systemic immune response.

### 3.13 Blood routine analysis

**Figure 7:**
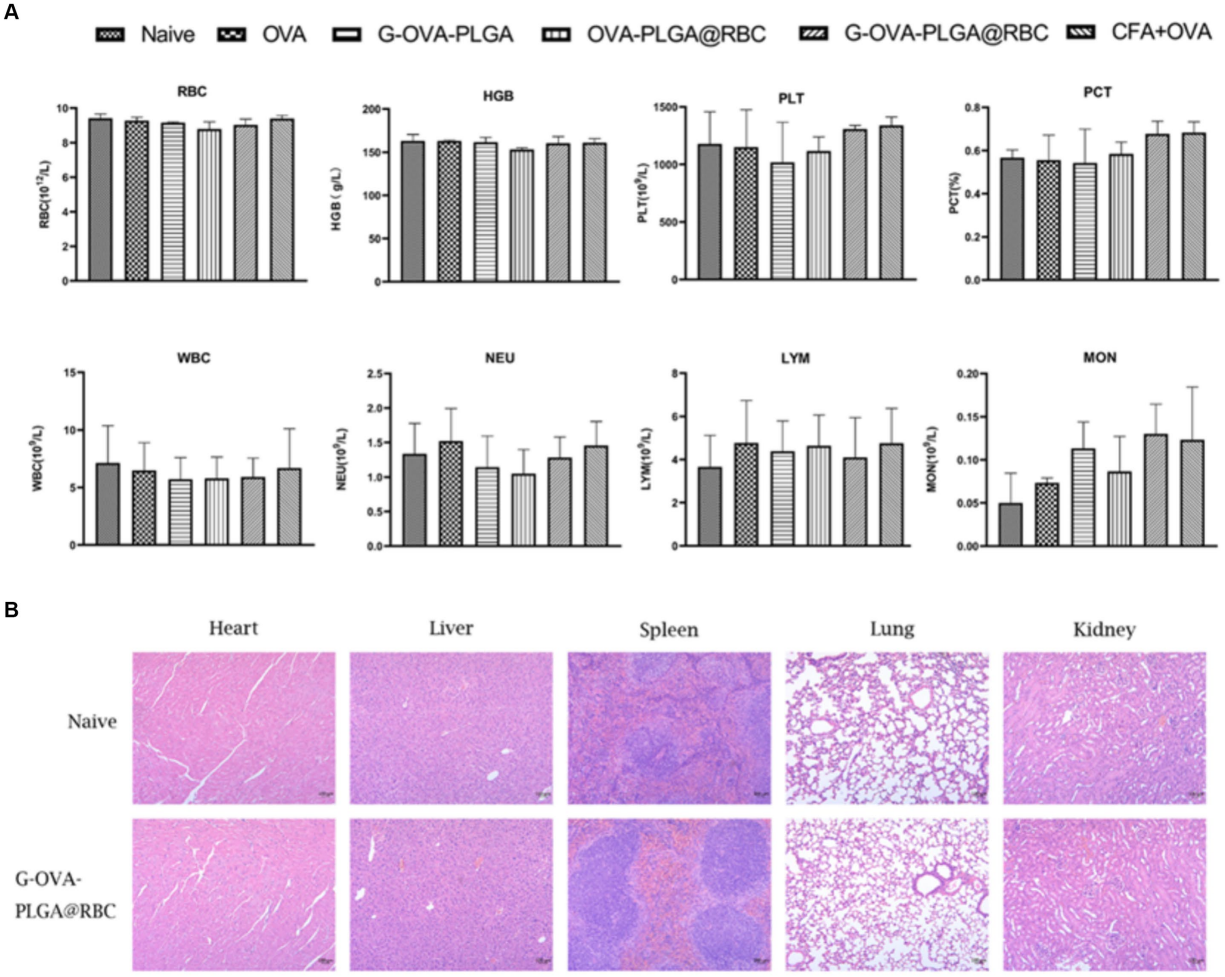
**(A)** Blood routine analysis, including RBCs, hemoglobin (HGB), platelets (PLT), platelets (PCT), white blood cells (WBC), neutrophils (NEU), lymphocytes (LYM), monocytes (MON). **(B)** Histopathological analysis of major organs (heart, liver, spleen, lung, kidney). The data has been expressed as the mean ± standard deviation (*n* = 3).

Blood constitutes a crucial component of animal body fluids (Figure 7A). In G-OVA-PLGA@RBC group, blood routine indicators, including RBC, HGB, PLT, PCT, WBC, NEU, LYM, and MON, exhibited no significant differences and remained within the normal range, indicating stability (*p* > 0.05). This indicates that the drug reagents possess favorable biocompatibility and are non-toxic.

### 3.14 Histopathological analysis

To evaluate the safety of G-OVA-PLGA@RBC in mice, the morphological changes in the heart, liver, spleen, lung, and kidney in the saline group and G-OVA-PLGA@RBC group were observed. As shown in Figure 7B, there were no lesions or inflammatory infiltration in the organs of the two groups, indicating that G-OVA-PLGA@RBC had good biocompatibility and safety.

## 4 Discussion

In recent years, β-glucan and its derivatives have been widely developed as vaccine adjuvants or vaccine delivery systems (31, 32). It is reported that after β-glucan is coupled to the E protein of Zika virus, the innate immune response of the E protein is amplified and adaptive immunity is generated (20). In addition, RBC-hitchhiking refers to a drug delivery system that binds drug-loaded nanoparticles to RBCs. This technology is based on the transient coupling of drug-loaded nanoparticles to red blood cells (1, 33). For example, RBCs transport drug-loaded antigen nanoparticles to antigen-presenting cells in the spleen and generate a strong humoral and cellular immune response (20). In this research, G-OVA-PLGA nanoparticles were prepared by coupling β-glucan and OVA through disulfide bonds, and the immune effect of the nanoparticles on macrophages was observed *in vitro* experiments. *In vivo* experiments, nanoparticles were loaded on the surface of red blood cells and injected into the body intravenously to observe the immune effect of the nanoparticles on the body.

Macrophages are very important immune cells in the immune system, destroying pathogens and foreign molecules through phagocytosis (34, 35). The activation status of macrophages can be divided into two categories: classic activated macrophages (M1) and alternative activated macrophages (M2) (36, 37). M1 can enhance the expression of MHC-II^+^, TNF-a, IL-1β, and IL-6, enhance the production of reactive oxygen species and NO, and have certain tumor damage activity (38, 39). M2 macrophages have anti-inflammatory and tumor-promoting effects (40). Previous studies have shown that β-glucan can exert immunomodulatory effects through Toll-like receptor 2 (TLR2), Complement receptor 3 (CR3) and Dectin-1. It not only has anti-inflammatory and pro-inflammatory effects, but also promotes the phagocytosis of macrophages (41). The data from this study confirmed that G-OVA-PLGA nanoparticles can increase the levels of CD80^+^ and CD86^+^ on the surface of macrophages, promote phagocytosis, and increase the production of NO, suggesting that G-OVA-PLGA can activate macrophages to enter the classical activated (M1) inflammatory state. In addition, *in vivo* experiments, G-OVA-PLGA@RBC not only significantly enhanced the expression of pro-inflammatory factors such as IL-6, IL-1β and IFN-γ, but also increased the expression levels of CD80^+^, CD86^+^, and MHC-II^+^ in dendritic cells.

Dendritic cells (DCs) play the role of core regulators in adaptive immune responses, are the most powerful professional antigen presenting cells in the body and are the only cell type that can effectively activate naive T cells (42–44). According to previous reports, immature DCs have extremely high ability to capture and process foreign substances, express various pathogen recognition receptors such as TLR on their surfaces, and constantly monitor and acquire dangerous signals in the surrounding environment (45). In addition, Dectin-1 receptors on dendritic cell membranes can recognize and capture β-glucans. Their recognition promotes cytokine production and dendritic cell maturation, and then enhances adaptive immune responses (46, 47). When these immature DCs transform into mature DCs, their surfaces significantly upregulate the expression of MHC molecules, surface stimulating molecules such as CD40^+^, CD80^+^ and CD86^+^ (48, 49). Therefore, the expression level of marker molecules on the surface of DC cells directly marks whether DC cells have matured and activated, which is a prerequisite for initiating an immune response (29). In this study, to understand the immune effect of G-OVA-PLGA and measure the maturity of dendritic cells, flow cytometry was used to measure the expression level of MHC-II^+^, CD80^+^, and CD86^+^ of DCs. The present results showed that G-OVA-PLGA nanoparticles enhance the expression of MHC-II^+^ molecules and co-stimulatory molecules CD80^+^ and CD86^+^ on the surface of dendritic cells, revealing that G-OVA-PLGA@RBC can effectively promote and stimulate the maturation of dendritic cells, thereby improving immunity.

RBC oxygenation serves as an independent sterilization mechanism, distinct from the functions of the liver and spleen (50). This mechanism is highly effective in eliminating bacteria within the bloodstream. Deceased bacteria are released back into the plasma and subsequently broken down and degraded within the cells of the reticuloendothelial system (RES) (51). The presence of the CD47 receptor on the surface of RBCs signals a “Do not eat me” message, allowing these cells to be recognized as self by the RES (52). This recognition enables the healthy RBCs to interact with the signal regulatory protein α on macrophages, facilitating their return to the bloodstream without being captured (51). In addition, RBCs have natural targeting capabilities, which can change the behavior of drug-loaded nanoparticles in the body and target drugs to specific sites, thereby extending the circulation time of drugs in the body (53, 54). In this experiment, the biodistribution of the G-OVA-PLGA@RBC group in organisms was evaluated through *in vivo* imaging observation. Within 0.5 h, the G-OVA-PLGA@RBC group had the highest fluorescence intensity in the lungs, and the red blood cells mounted on the nanoparticles were subjected to shear stress when passing through the pulmonary capillaries (50). Therefore, the nanoparticles fell off from the red blood cells and stayed in the lungs. Over the next 24 h, the fluorescence intensity in the spleen and liver of the G-OVA-PLGA@RBC group was significantly higher than that of the G-OVA-PLGA and control groups, and the nanoparticles gradually entered the spleen and liver, where they were captured by the reticuloendothelial tissue (33). Experimental results show that RBC-hitchhiking effectively changes the biological distribution of G-OVA-PLGA nanoparticles in the body. This change significantly increased the circulation time of nanoparticles and prolonged the residence time of OVA and β-glucan in the body. In addition, G-OVA-PLGA showed slow-release characteristics *in vitro* release experiments. These resulted in the production of high levels of specific IgG antibodies in mice, indicating that G-OVA-PLGA@RBC could promote an effective and durable immune response.

The present study showed that the delivery system can improve the delivery efficiency of antigens and adjuvants and enhance the immune response *in vivo* and *in vitro*. The current preliminary exploration of the way in which RBC-hitchhiking can quickly deliver antigens and adjuvants into the body has great potential, and the potential application of the RBC-hitchhiking drug delivery system in other animal models will be further explored in the future.

## Data Availability

The original contributions presented in the study are included in the article/supplementary material, further inquiries can be directed to the corresponding author.
